# Evaluating the suitability of current mitochondrial DNA interpretation guidelines for multigenerational whole mitochondrial genome comparisons

**DOI:** 10.1111/1556-4029.15097

**Published:** 2022-07-19

**Authors:** Jasmine R. Connell, Miles C. Benton, Rodney A. Lea, Heidi G. Sutherland, Larisa M. Haupt, Kirsty M. Wright, Lyn R. Griffiths

**Affiliations:** ^1^ Queensland University of Technology (QUT) Centre for Genomics and Personalised Health Genomics Research Centre School of Biomedical Sciences Kelvin Grove Qld Australia; ^2^ Unrecovered War Casualties‐Army Australian Defence Force, Russell Offices Russell ACT Australia; ^3^ Royal Australian Air Force (RAAF) Headquarters History and Heritage Unrecovered War Casualties‐Air Force Russell ACT Australia; ^4^ Human Genomics Kenepuru Science Centre Institute of Environmental Science and Research Wellington New Zealand

**Keywords:** DNA analysis, DNA Commission of the International Society of Forensic Genetics, heteroplasmy, historical casework, historical military identification, historical military remains, historical remains, human identification, ISFG, mitochondrial DNA interpretation guidelines, mtDNA interpretation guidelines, Scientific Working Group of DNA Analysis Methods, SWGDAM, whole mitochondrial genome, whole mtGenome

## Abstract

Sanger sequencing of the mitochondrial DNA (mtDNA) control region was previously the only method available for forensic casework involving degraded samples from skeletal remains. The introduction of Next Generation Sequencing (NGS) has transformed genetic data generation and human identification using mtDNA. Whole mitochondrial genome (mtGenome) analysis is now being introduced into forensic laboratories around the world to analyze historical remains. Research into large pedigrees using the mtGenome is critical to evaluate currently available interpretation guidelines for mtDNA analysis, which were developed for comparisons using the control region. This study included mtGenomes from 225 individuals from the last four generations of the Norfolk Island (NI) genetic isolate pedigree consisting of 49 distinct maternal lineages. The data from these individuals were arranged into 2339 maternally related pairs separated by up to 18 meioses. Our results show that 97.3% of maternally related pairs were concordant at all nucleotide positions, resulting in the correct interpretation of “Cannot Exclude”; 2.7% of pairs produced an “Inconclusive” result, and there were no instances of false exclusion. While these results indicate that existing guidelines are suitable for multigenerational whole mtGenome analysis, we recommend caution be taken when classifying heteroplasmic changes as differences for human identification. Our data showed the classification of heteroplasmic changes as differences increases the prevalence of inconclusive identification by 6%, with false exclusions observed in 0.34% of pairs examined. Further studies of multigenerational pedigrees, however, are needed to validate mtGenome interpretation guidelines for historical case work to more fully utilize emerging advancements.

**Highlights**

- Differences between maternal pairs (including heteroplasmy) were confirmed at 9 positions across the mtGenome.
- Classifying heteroplasmic changes as differences led to 8 false exclusions (0.34%).
- No instances where maternally related individuals differed at two or more nucleotide positions.
- Existing interpretation guidelines are suitable for multigenerational whole mtGenome analysis.
- No difference should be stated if heteroplasmic base matches homoplasmic type in comparison sample.

## 1 INTRODUCTION

The mitochondrial genome (mtGenome) is a small double‐stranded genome that is split into two sections: a large coding region responsible for gene production for transfer RNA, ribosomal RNA translation, and cellular energy production; and a smaller control region [1]. The high copy number of mtDNA in each cell means that it is often recoverable when nuclear DNA is significantly degraded [2, 3].

The interpretation and evaluation of mtDNA sequencing results is the final step in a complex analysis process, and factors such as heteroplasmy, nomenclature ambiguities, database searches, and mtDNA mutation rate can make the interpretation more arduous. In extreme instances, these ambiguities may result in different conclusions, and it is, therefore, critical to understand all aspects of the analysis process to ensure reliable interpretation of the results. The international community has attempted to address these problems through various guidelines and recommendations. While the Scientific Working Group on DNA Analysis Methods (SWDAM) [4] and DNA Commission of the International Society of Forensic Genetics (ISFG) are the main providers [5, 6, 7, 8], other guidelines also exist [9, 10]. With technological advances and as additional resources become available, these guidelines are often revised to accurately reflect the state of the field at the time.

While most forensic laboratories perform Sanger sequencing (SS) for the entire control region as a routine methodology [11, 12, 13, 14], these regions may not provide sufficient discrimination power for forensic purposes [1, 15, 16, 17, 18, 19, 20, 21]. Since first introduced in 2005, the advantages of Next Generation Sequencing (NGS) have been exploited for forensic casework, and unsurprisingly, researchers have used this technology to sequence the entire mtGenome with the intent of improving human identification [1, 16, 17, 18, 19, 20, 22, 23, 24]. In 2019, revisions to the SWDAM interpretation guidelines for mtDNA analysis (referred to hereafter as the SWGDAM guidelines) were approved, and now address NGS data analysis [4]. At present, however, one of the greatest barriers with the wider implementation of whole mtGenome sequencing in forensic casework is the uncertainty of appropriate interpretation guidelines. The current SWGDAM guidelines were established for use on the mtDNA control region with comparisons involving close relatives, acknowledging that they may need to be modified when reference samples are from distant maternal relatives, or when the sequences compared extend beyond the control region [4]. ISFG guidelines do not currently provide specific information on these limitations.

Previous studies have begun to explore the possibility of single‐meiosis differences across the mtGenome, focusing on the transmission of heteroplasmic variants. For example, Ma et al. demonstrated that mtDNA variants across the entire mtGenome were inherited without exception within mother–child pairs, but with different frequencies observed per individual. Authors focused on heteroplasmic variants with a minor allele frequency (MAF) >10% [25]. Other studies, such as Zaidi et al., examined heteroplasmy transmission in multigenerational families, concluding that mutation frequencies can change dramatically between mother–child pairs [26]. Until now, studies examining the suitability of existing guidelines for entire mtGenome comparisons involving distant relatives have been lacking.

This research therefore aimed to evaluate the suitability of the current guidelines: a) for comparisons involving distant relatives (multigenerational analysis) and b) where analysis includes the entire mtGenome. This research focused on the combined use of both SWGDAM and ISFG guidelines. We investigated the complete mtGenome from 225 individuals from the multigenerational Norfolk Island (NI) pedigree, resulting in 2339 maternally related pairs from the last four generations of the core pedigree [27]. The use of the NI pedigree provided an opportunity to construct a large number of descendant pairs across a range of meioses consistent with historical casework. The entire mtGenome was interrogated to determine the frequency and location of any nucleotide differences within the maternal pairs. With these data, we addressed a) the hypothesis that sequencing the entire mtGenome introduces additional variation between maternally related pairs than what is observed when sequencing the control region, and b) the number of meiotic events affects the percentage of nucleotide differences observed between two maternally related pairs. The results of these two hypotheses led to modified guidelines (recommendations), which are outlined in this manuscript. The final hypothesis in this research is that applying these modified recommendations leads to a reduction in the false exclusion rate.

## 2 METHODS

### 2.1 Sample selection

The sample information for this research was described previously [28]. Individuals chosen for this research were included in the NI Health Study and the associated NI core pedigree for research investigations at Queensland University of Technology. The NI Health Study has been well described in previous research [27, 29, 30, 31, 32]. All individuals chosen for this research were from the last four generations of the NI core pedigree. For illustration, the NI core pedigree is shown in Figure S1A. Using the NI core pedigree, individual maternal pedigrees were constructed by establishing a list of founding mothers and tracing their maternal line. Pedigree construction and analysis has been described previously [28]. In total, 45 pedigrees (families) were chosen (Figure S1B). From these families, 225 individuals (including 125 females and 100 males) were chosen, corresponding to 345 mtDNA transmissions and 2339 maternally related pairs. The number of pairs per meiotic category is outlined in Table 1.

**TABLE 1:** Number of maternally related pairs per meiotic category that separate them

Meiotic category	Count	Percentage
1	71	3.0
2	94	4.0
3	72	3.1
4	73	3.1
5	67	2.9
6	55	2.4
7	41	1.8
8	51	2.2
9	90	3.8
10	149	6.4
11	265	11.3
12	316	13.5
13	339	14.5
14	323	13.8
15	211	9.0
16	96	4.1
17	18	0.8
18	8	0.3

*Note*: Total sample size *n* = 2339.

All participants provided informed consent for research involvement. Ethical clearance for the NI mitochondrial DNA analysis portion of this study was provided originally by the Griffith University Human Research Ethics Committee (Approval MSC/04/09/HREC). Ethical clearance was transferred to and is now provided by the Queensland University of Technology Human Research Ethics Committee (Approval Number: 1400000749). No other ethical clearance was required.

### 2.2 Library preparation and sequencing

Sequencing of these individuals was performed in earlier work as previously outlined [33]. In brief, amplification of the entire mtGenome involved using long‐range PCR with two overlapping primer sets. Library preparation and sequencing was performed using an Ion Torrent high throughout sequencing protocol established in‐house [33].

### 2.3 Data analysis

**FIGURE 1:**
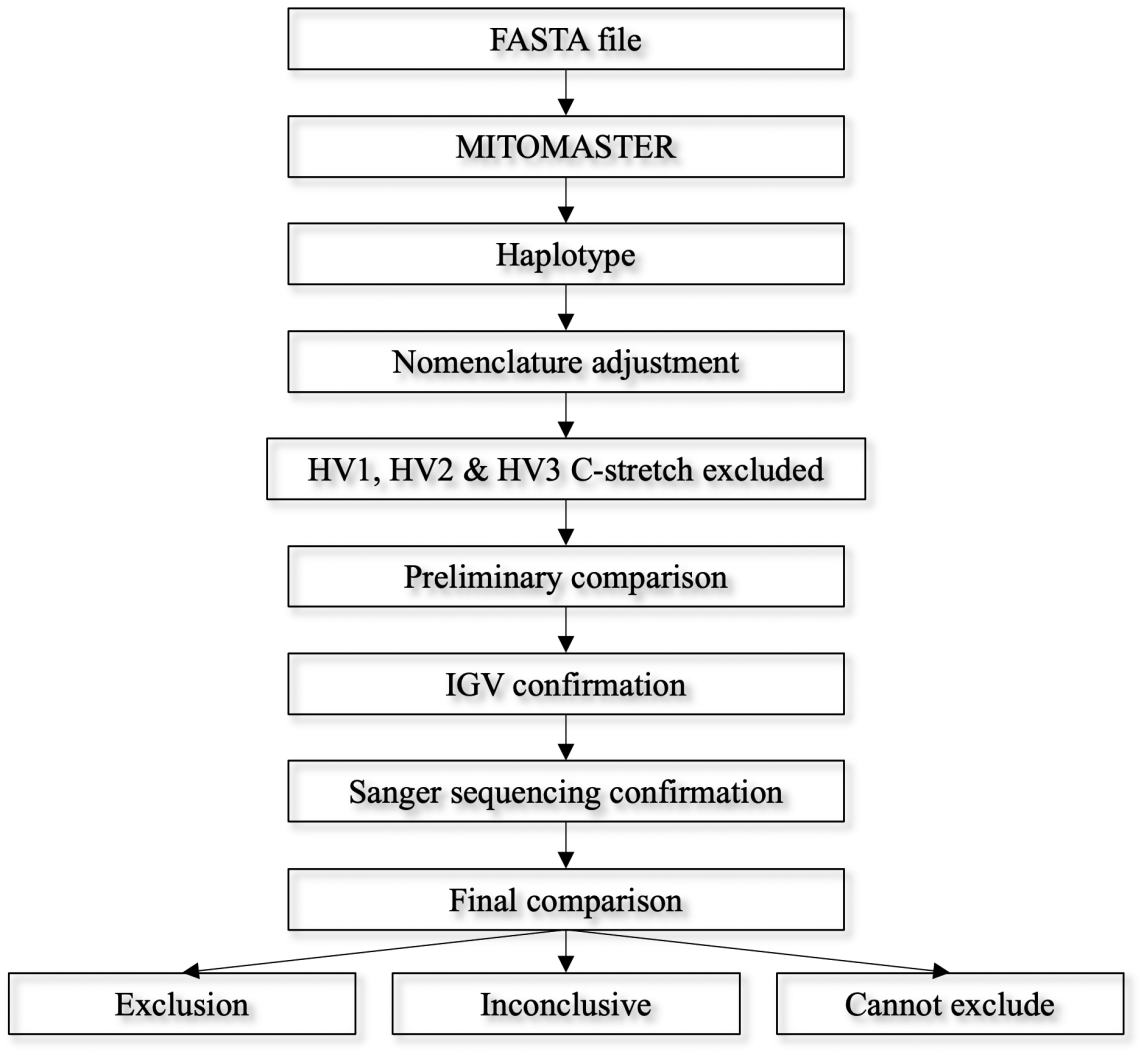
Overview of the interpretation process. The process undertaken to identify mtDNA haplotypes for each participant and perform sequence comparisons. Haplotypes are generated using MITOMASTER, then altered to align with existing interpretation guidelines. Variants were confirmed through SS before undertaking final comparison

FASTA files were generated using an in‐house bioinformatics pipeline outlined in Harvey et al. [33] and uploaded to MITOMASTER [34] for alignment against the revised Cambridge Reference Sequence (rCRS) to classify nucleotide variants (haplotypes) for each participant (Figure 1). Where required, haplotypes were adjusted to ensure they followed SWGDAM and ISFG nomenclature guidelines. For example, insertions and deletions were moved to the 3′ end of the light strand.

While literature indicates that NGS is valid for the quantification of heteroplasmy >1% [35], this lower limit often requires independent DNA extraction, PCR amplification, and sequencing to authenticate heteroplasmy results and exclude contamination [36, 37, 38]. Furthermore, such variants are often undetected in SS trace data, where the detection limit is typically described as 10%–20% [39, 40, 41]. Additional samples were unavailable for this research with a conservative minor allele frequency (MAF) of >20% utilized to reduce incorrect heteroplasmy calls. Heteroplasmy was determined as described in Connell et al. [28], using the MAF, the peak height in electropherograms, and the reproducibility of peaks.

The poly‐C tracks of both HVI and HVII are known to have high indel rates, introducing length heteroplasmy [42]. For example, length heteroplasmy in the poly‐C tract of HVI and HVII occurred in 22.9% and 54.2%, respectively [36]. The detection of length heteroplasmy depends on the technology used, and the distribution varies within and between tissues of an individual [43, 44, 45]. As per SWGDAM guidelines, no attempt was made to determine the exact number of bases in an HV1 C‐stretch between positions 16,183 and 16,194. Furthermore, both SWGDAM and ISFG guidelines indicate that laboratories must establish their own interpretation guidelines for heteroplasmy. As such, no attempt was made to determine the exact number of cytosine bases in the HV2 C‐stretch region between positions 302 and 310, or the HV3 C‐stretch between 568 and 573. All comparisons assumed the same number is present. Point and length heteroplasmy at other locations within the mtGenome were reported in the haplotypes.

The haplotypes of maternally related individuals were compared to establish a preliminary list of differing variants (Figure 1). No minimum read count or coverage threshold was required for variant calling at this stage. Where possible, these differences were verified using the Integrative Genomics Viewer (IGV) tool [46]. Base differences and heteroplasmy (excluding hypervariable region C‐stretches) observed between maternal pairs were confirmed using SS via the BigDye Terminator v3.1 Cycle Sequencing Kit on the 3500 Genetic Analyzer (Thermo fisher Scientific). Methods and primers used for SS were described previously [28]. Sanger sequencing primers are outlined in Table S1. Ideally, sequencing was performed in both forward and reverse direction, with some sample sequences confirmed twice in the same direction due to difficulties with primer design for sequencing. Review of the SS results included a comparison of the variants generated by two independent scientists. No discrepancies were obtained between the two comparisons. Following SS, a final sequence comparison was performed using SWGDAM guidelines of:

1. Exclusion: When samples differed at two or more nucleotide positions, they were excluded as coming from the same source or maternal lineage
2. Inconclusive: When samples differed at a single position only, the result was deemed inconclusive
3. Cannot Exclude: When samples shared a common DNA base at every nucleotide position, they could not be excluded as coming from the same maternal lineage.

Sequence comparisons for each maternally related pair were performed twice. The first as per the ISFG guidelines, where differences in point or length heteroplasmy were not evidence for excluding two otherwise concordant haplotypes as deriving from the same maternal lineage. A second comparison was performed that included instances of point and length heteroplasmy across the mtGenome, except those within the hypervariable region C‐stretches. The relationship between the number of observed differences across the entire mitochondrial genome and the number of meioses that separate two maternally related individuals was also examined.

Confidence intervals (CIs: 95%) for the guideline interpretations were calculated using Epitools, an online tool provided by AusVet Animal Health Services [47]. The program outputs intervals using five alternative calculation methods as described in Brown et al. [48]. In accordance with SWGDAM guidelines [4], the Clopper and Pearson method was used for this research. However, since this method has been reported as overly conservative and inefficient [49], intervals were also reported using the Wilson method. Chi‐squared and Fisher exact tests were performed using R studio.

## 3 RESULTS AND DISCUSSION

### 3.1 Sample information and sequence quality

Our study included 225 individuals from the last four generations of the NI core pedigree, resulting in 2339 maternal pairs. The individuals in these pairs are separated by up to 18 meioses. Output sequencing produced approximately 2.5 × 10^6^ 200 bp‐single‐end reads per Ion 316 chip. The sequencing coverage across the mitochondrial genome reached a median depth of ~370X (minimum: 139X; maximum: 1316X) for all 225 samples. Sequence quality (Phred) scores remained consistent at >25 for all samples at the median read length (140 bp). The per sequence guanine‐cytosine (GC) content followed normal distribution as expected for NGS data. The haplogroup and number of heteroplasmic sites per sample (excluding the HV1 C‐stretch positions 16,183–16,194, the HV2 C‐stretch positions 302–310, or the HV3 C‐stretch between 568 and 573) are defined in Table S2.

### 3.2 Sequence comparisons

**FIGURE 2:**
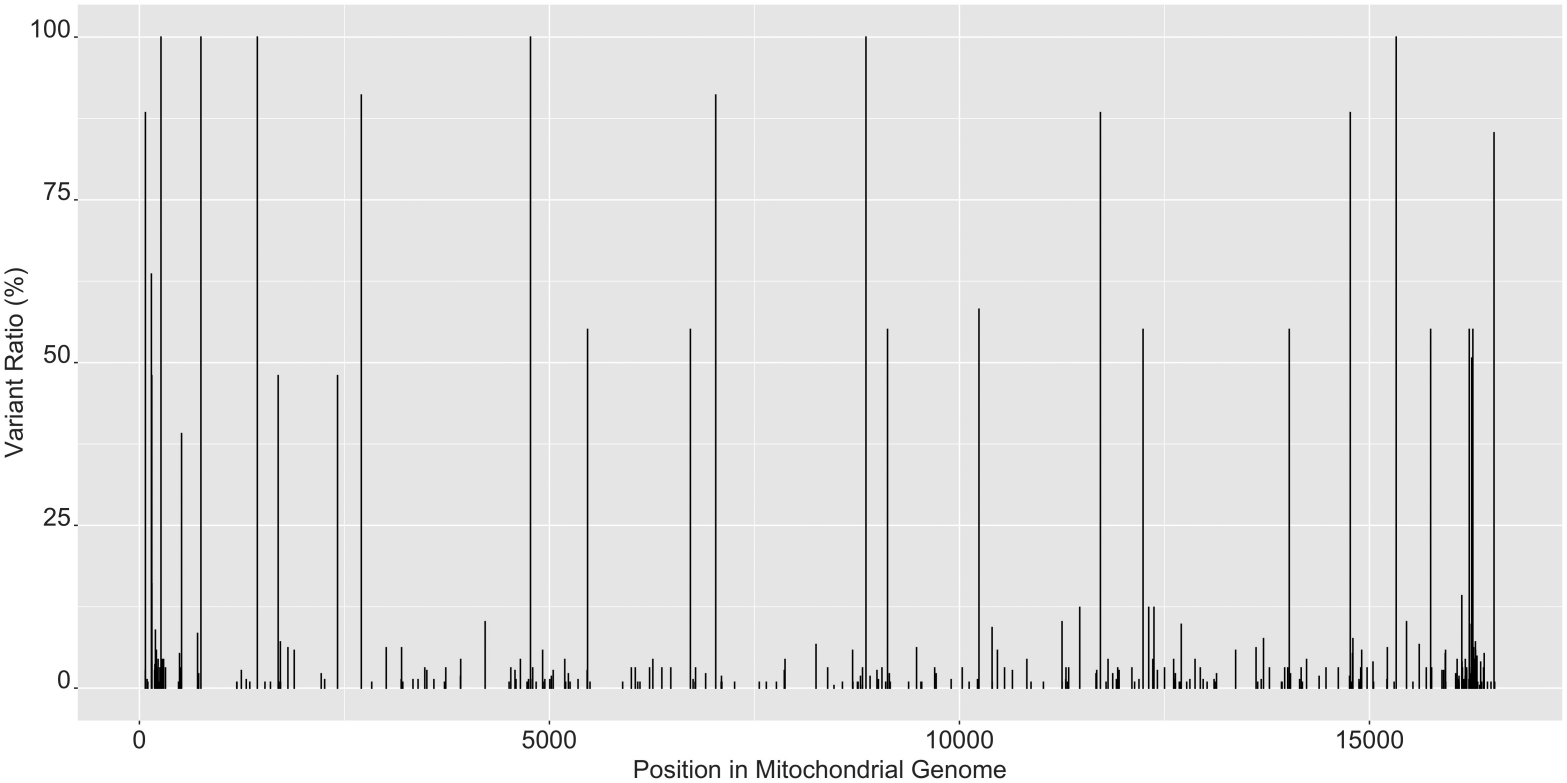
Map of variants across the mitochondrial genome. Map showing the frequency for variants observed across the entire mitochondrial genome from 225 individuals from Norfolk Island. Excludes variants within the hypervariable regions I, II, and III C‐stretch

The mtGenome haplotypes of maternally related pairs were then compared to determine the frequency and location of any differing variants and to test the hypothesis that the sequencing the entire mtGenome introduces additional variation between maternally related pairs than what is observed when sequencing the control region. Differences between maternal pairs (including heteroplasmy) were confirmed at 9 positions across the mtGenome (Table 2) and were confined to 7 of the 45 (15.55%) maternal lineages studied. We found that 70.7% of all variants were located within the coding region (Figure 2), and the number of observed differences between maternal pairs increased by 87.5% when sequencing the entire mtGenome compared to the control region (including heteroplasmic changes). This supports our hypothesis that sequencing the entire mtGenome introduces additional variation between maternally related pairs.

**TABLE 2:** Location of differing variants observed in 2339 maternally related pairs across the entire mitochondrial genome

Position^a^	Region	Type	Count per maternal lineage
T146Y	HVII (Control region)	Point heteroplasmy	1
A2833R	Coding region	Point heteroplasmy	2
A8470R	Coding region	Point heteroplasmy	1
A8817G	Coding region	Substitution	1
T9012Y	Coding region	Point heteroplasmy	3
A16247G	HVI (Control region)	Substitution	1
A16280R	HVI (Control region)	Point heteroplasmy	3
C16320Y	HVI (Control region)	Point heteroplasmy	2
C16344Y	HVI (Control region)	Point heteroplasmy	1

^a^ DNA bases are designated by the nomenclature system set forth by the International Union of Pure and Applied Chemistry (IUPAC).

The frequency of point heteroplasmy in the NI sample was not associated with any particular mtDNA haplogroup (Fisher exact test: *p* = 0.080). No length heteroplasmy was observed, other than in hypervariable region C‐stretches, where no attempt was made to determine the exact number of bases. As per ISFG guidelines, differences in point or length heteroplasmy were not evidence for excluding two otherwise concordant haplotypes as deriving from the same maternal lineage. Hence, heteroplasmic differences were not considered in the following summary. Across 45 families, there were 2275 (97.3%) maternally related pairs, separated by 1 to 18 meioses, who shared a common DNA base at every nucleotide position along the mtGenome. Individuals in 2.7% of maternally related pairs differed at a single nucleotide position, which was the result of variants in two individuals across 64 maternal pairs from two separate pedigrees (Family 8 and Family 12). There were no instances where maternally related individuals differed at two or more nucleotide positions.

#### 3.2.1 Family 8

The entire mtGenome was sequenced for seven maternally related individuals from Family 8 (two women and five men), which revealed an A16247G variant in HVI in one individual (Figure S2B,D). Individuals were concordant at all other positions in the mtGenome. Sequencing results for a representative family member are provided in Figure S2C,E.

#### 3.2.2 Family 12

The entire mtGenome was sequenced for 59 maternally related individuals from Family 12 (35 females and 24 males), which revealed a homoplasmic A8817G variant in the mitochondrial *MT‐ATP6* gene in one individual (Figure S3B,D). The presence of the variant was confirmed by SS (Figure S3D). Point heteroplasmy was also observed in two individuals from Family 12 (MAF 39% A and 27% G, respectively) at position 2833 in the MT‐RNR2 gene (Figure S3A, black arrows), however heteroplasmic differences were not considered for sequence comparisons. Point heteroplasmy in both individuals was validated by SS (data not shown). The remaining members of the pedigree were concordant with the rCRS and showed no signs of heteroplasmy.

**FIGURE 3:**
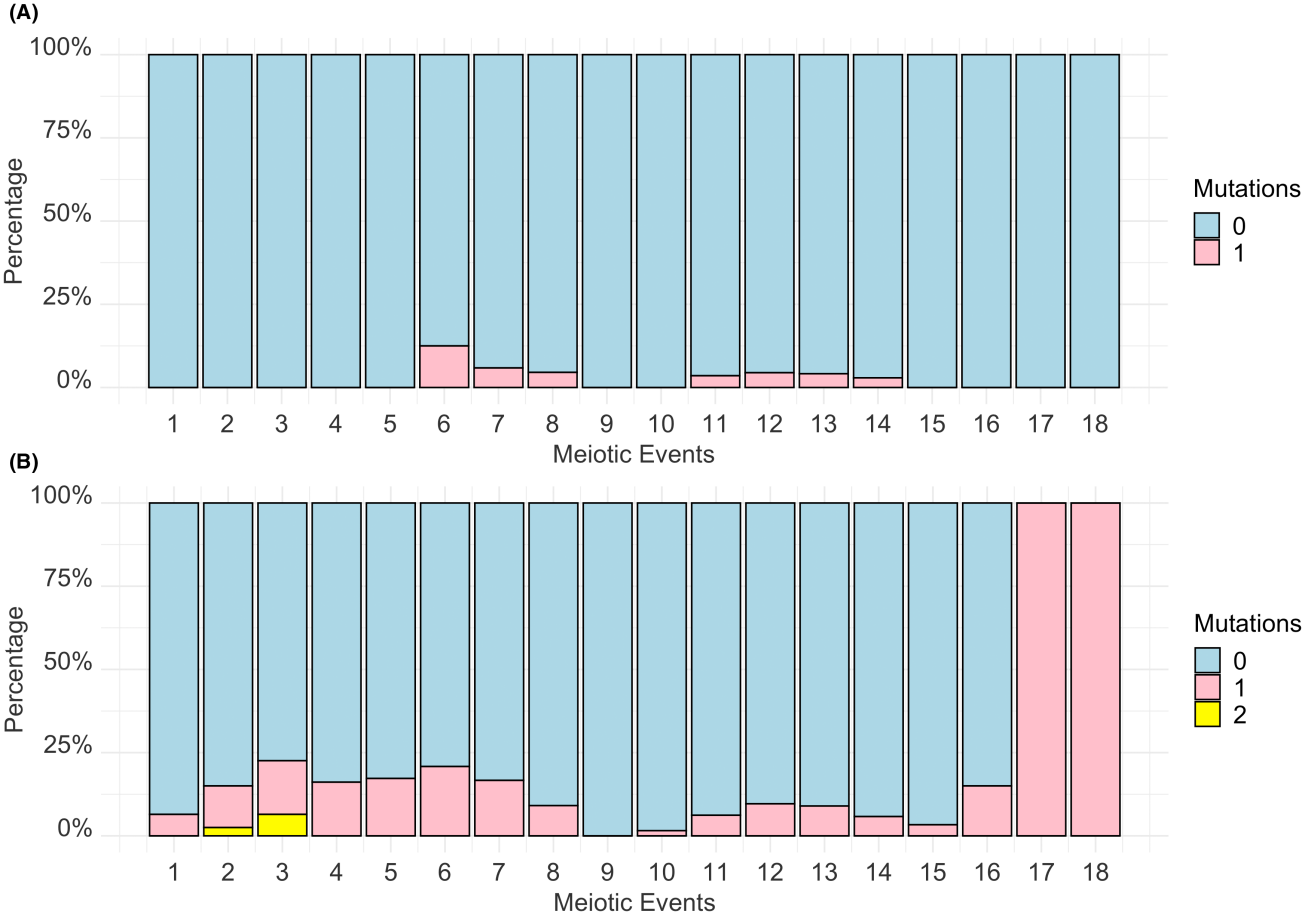
Differing variants observed between maternal pairs in the Norfolk Island sample, *n* = 2339. Graphical representation showing the relationship between the number of differing variants across the entire mitochondrial genome and the number of meioses that separate two maternally related individuals who are separated by up to 18 meioses. (A) No relationship is observed when heteroplasmic changes are not classified as differences. (B) Heteroplasmic changes are classified as differences. All maternally related pairs separated by more than 16 meiotic events differed by one heteroplasmic change (*n* = 122)

Figure 3 shows the number of differing variants observed between maternally related pairs with reference to the number of meiotic events that separate them. When heteroplasmic changes were not classified as differences, no relationship was observed between the occurrence of differing variants across the entire mtGenome relative to the number of meioses between the maternally related pairs (Figure 3A). When heteroplasmic changes are classified as differences, our results show an overall increase in the number of differences between maternally related pairs. Interestingly, all maternally related pairs separated by more than 16 meiotic events differed by one heteroplasmic variant (*n* = 122) (Figure 3B). This could be due to limitations with sample size for maternally related pairs with more than 16 meiotic events between them or could be due to the degree of separation between these pairs. These results appear to support our hypothesis that the number of meiotic events affects the percentage of nucleotide differences observed between two maternally related pairs and raise the question of whether heteroplasmic changes should be classified as differences for human identification purposes.

While the conservative MAF threshold of >20% was chosen to reduce incorrect heteroplasmy calls, upon further examination of the positions detailed in Table 2, the NGS threshold was too high for calling the observable heteroplasmy in SS chromatograms for eight individuals. For example, one individual from Family 12 presented with a A8817G substitution (MAF 8% A, well below the detection threshold), despite the evidence of heteroplasmy in the SS chromatogram. In this example, if the A8817G substitution was reported as heteroplasmy, the number of inconclusive comparisons overall would decrease from 2.74% to 0.26%. It is important to note that this reduction is only true under the assumption that when one of the heteroplasmic bases matches the homoplasmic type in the comparison sample, no difference is reported. Reduction of the detection threshold to 5% would be required to allow for correct detection of heteroplasmy in all eight samples, however this would also introduce a false positive result for two individuals. Furthermore, reduction of the detection threshold to 5% may introduce further heteroplasmic sites not detected with the 20% threshold set.

### 3.3 Evaluation of existing guidelines

Existing guidelines for mtDNA interpretation using the control region utilize a count method, where two or more differences between comparison samples result in an *Exclusion*, that is, samples can be excluded as coming from the same source or maternal lineage. We identified no false exclusions in 2339 maternally related pairs separated by up to 18 meioses (Table 3a) and therefore, our data support the use of existing guidelines: a) for comparisons involving distant relatives (multigenerational analysis) and b) when analysis includes the entire mtGenome. However, our results rely on the understanding that differences in heteroplasmy are not enough evidence for excluding two otherwise identical haplotypes as originating from the same maternal lineage or source. While this is outlined in Recommendation #10 of ISFG guidelines (and others, e.g., [9]), it is not unambiguously stated in SWGDAM guidelines. Furthermore, both SWGDAM and ISFG guidelines also state that laboratories should develop their own guidelines for the evaluation of cases involving heteroplasmy [4, 5, 6, 7, 8]. Caution should be taken when classifying heteroplasmic changes as differences for human identification. NGS is a cost‐effective, high‐throughput, and sensitive method that allows for the detection of any DNA variants, even if they are present at low levels. Therefore, this method is superior to SS for identification of heteroplasmy [50, 51, 52], and can detect variants with a frequency lower than 1% and even 0.5% [35, 53]. Other studies have identified that the frequency of heteroplasmy can differ across tissue types [44, 45], introducing further issues with the classification of heteroplasmy as a difference in human identification casework, particularly where the source of DNA varies between the donor and reference sample. If laboratories classify heteroplasmy as a difference in sequence comparisons, we suggest the following recommendation:

**TABLE 3:** Differing variants observed in the entire mtGenome across 2339 maternally related pairs

Outcome^a^	Count	Percentage	95% Confidence interval	
			Clopper Pearson	Wilson
a) Differences in heteroplasmy were evidence for excluding two otherwise concordant haplotypes.				
Cannot exclude	2275	97.26	96.52–97.89	96.52–97.85
Inconclusive	64	2.74	2.11–3.48	2.15–3.48
Exclude	0	0.00	0.00–0.16	0.0–0.16
b) Differences in heteroplasmy were not evidence for excluding two otherwise concordant haplotypes				
Cannot exclude	2114	90.38	89.11–91.55	89.12–91.51
Inconclusive	217	9.28	8.13–10.53	8.17–10.52
Exclude	8	0.34	0.15–0.67	0.17–0.67

^a^ Outcome uses terminology outlined in the Scientific Working Group on DNA Analysis Methods Interpretation Guidelines for Mitochondrial DNA Analysis by Forensic DNA Testing Laboratories [4].

Where one of the heteroplasmic bases matches the homoplasmic type in the comparison sample, no differences should be reported.

One of the heteroplasmic bases matched the homoplasmic type in the comparison sample in 163 (6.97%) maternally related pairs from our NI sample. If this recommendation was not considered, an inconclusive result would be returned in almost 10% of the maternally related pairs examined, and a false exclusion reported in eight cases (0.34%) (Table 3b). This recommendation significantly reduced the number of false exclusions reported for sequence comparisons compared to analysis performed with the classification of heteroplasmic changes as differences (Chi‐squared test: X^2^ = 97.212, df = 2, *p < 2.2x10* ^*−16*^). This supports our third and final hypothesis and highlights the need for detailed consideration when qualifying heteroplasmic changes as differences for human identification purposes. There were no instances where the homoplasmic base was inconsistent with both heteroplasmic bases in the comparison sample.

Although extensive, the NI pedigree alone is not sufficient to address the suitability of current mtDNA interpretation guidelines. As such, we propose further collaboration between health and medical researchers and forensic scientists. Several large pedigrees exist that would prove beneficial for examining the suitability of mtDNA interpretation guidelines (if appropriate consent exists). Collectively, existing whole mtGenome data from large extended pedigrees should be explored for a better understanding of private intergenerational mutation rates and comprehensive validation of multigenerational interpretation guidelines. This research is paramount to prevent misidentification or false exclusion, and thus for the accurate identification of historical remains. Well‐characterized large pedigrees that already have good quality sequence data, for example for health and medical research purposes, provide an efficient use of resources, rather than forensic science researchers establishing new pedigrees at great cost.

## 4 CONCLUSION

This study provides the first evaluation of current guidelines for use with extended pedigree analysis encompassing the entire mtGenome. When heteroplasmic changes are not classified as differences (as per existing guidelines), our data found no relationship between the occurrence of differing variants across the entire mtGenome relative to the number of meioses between the maternally related pairs examined. We identified no false exclusions in 2339 maternally related pairs separated by up to 18 meioses, and therefore our data supports the use of existing guidelines for human identification involving the entire mtGenome. However, while both SWGDAM and ISFG guidelines indicate laboratories must establish their own interpretation guidelines for heteroplasmy, we recommend caution be taken when qualifying heteroplasmic changes as differences for human identification as the counting of heteroplasmic differences increased the prevalence of inconclusive identification by 6%, and false exclusions were observed in 0.34% of pairs examined. Greater accuracy in mtGenome interpretation methods will reduce the risk of incorrect identification and improve valid identification of historical remains. Our findings have application and implications for various groups, including those investigating historical military remains including Unrecovered War Casualties—Army (Australia) and Armed Forces DNA Identification Laboratory (United States), and criminal and coronial investigations involving long‐term missing persons.

## FUNDING INFORMATION

The Norfolk Island mitochondrial analysis was supported by NHMRC grants 376608, 536518 and 1058806 (LRG). This research was also supported by infrastructure purchased with Australian Government EIF Super Science Funds as part of the Therapeutic Innovation Australia–Queensland Node project (LRG). In addition, Jasmine Connell was the recipient of an Institute of Health and Biomedical Innovation (IHBI) Queensland University of Technology (QUT) postgraduate student scholarship and Miles Benton was supported by a Corbett Postgraduate Research Grant.

## CODE AVAILABILITY

The code utilized for this study is available in the GitHub repository https://github.com/GRC‐CompGen/mitochondrial_seq_pipeline and http://sirselim.github.io/presentations/mt_tracing.html

## ETHICAL APPROVAL

Ethical clearance for the Norfolk Island mitochondrial DNA analysis portion of this study was provided originally by the Griffith University Human Research Ethics Committee (Approval MSC/04/09/HREC). Ethical clearance was transferred to and is now provided by the Queensland University of Technology Human Research Ethics Committee (Approval Number: 1400000749). No other ethical clearance was required.

## CONSENT TO PARTICIPATE

All Norfolk participants provided informed consent for research involvement.

## Supporting information


Figure S1

Figure S2

Figure S3

Table S1

Table S2
