# Supplementary material for: Evaluating the suitability of current mitochondrial DNA interpretation guidelines for multigenerational whole mitochondrial genome comparisons
Source: J Forensic Sci. 2022 Jul 19;67(5):1766–75. doi: 10.1111/1556-4029.15097 (PMC9543078; doi:10.1111/1556-4029.15097)
Supplement: Supplementary file 1 — Figure S1 [file JFO-67-1766-s001.docx]

FIGURE S1 Norfolk Island (NI) core and resultant pedigrees.

a)

b)


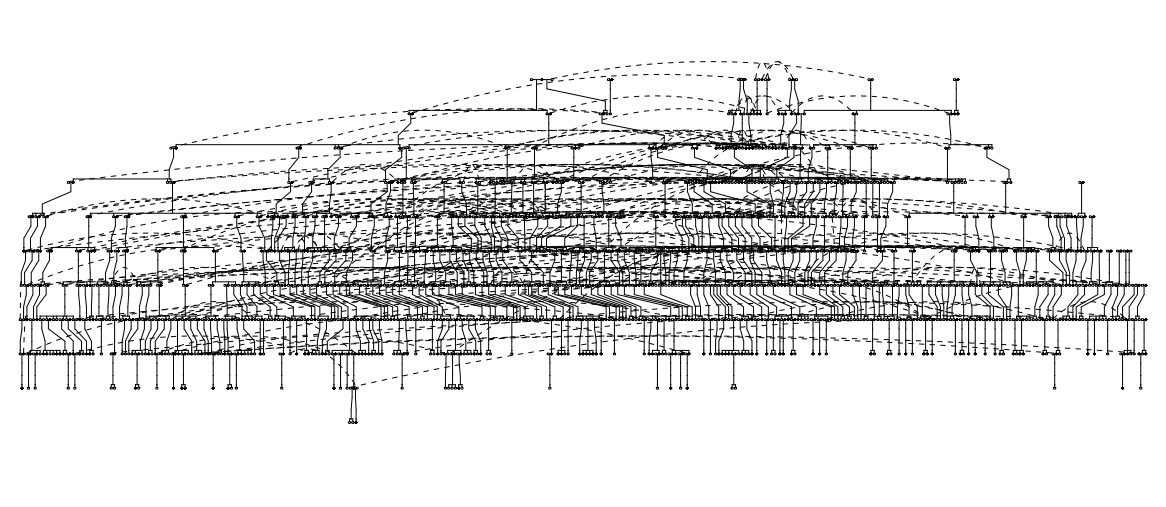

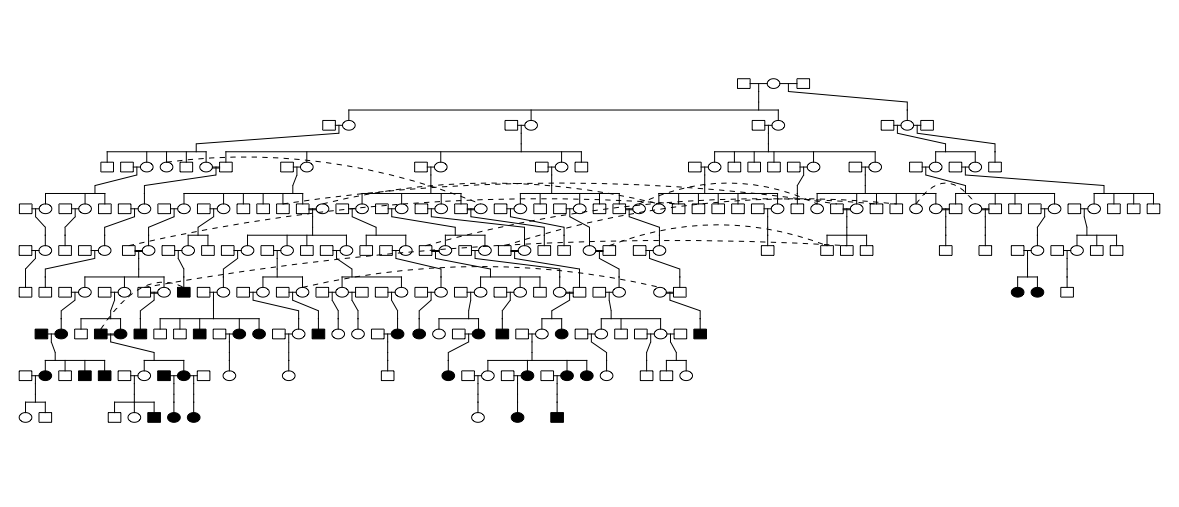

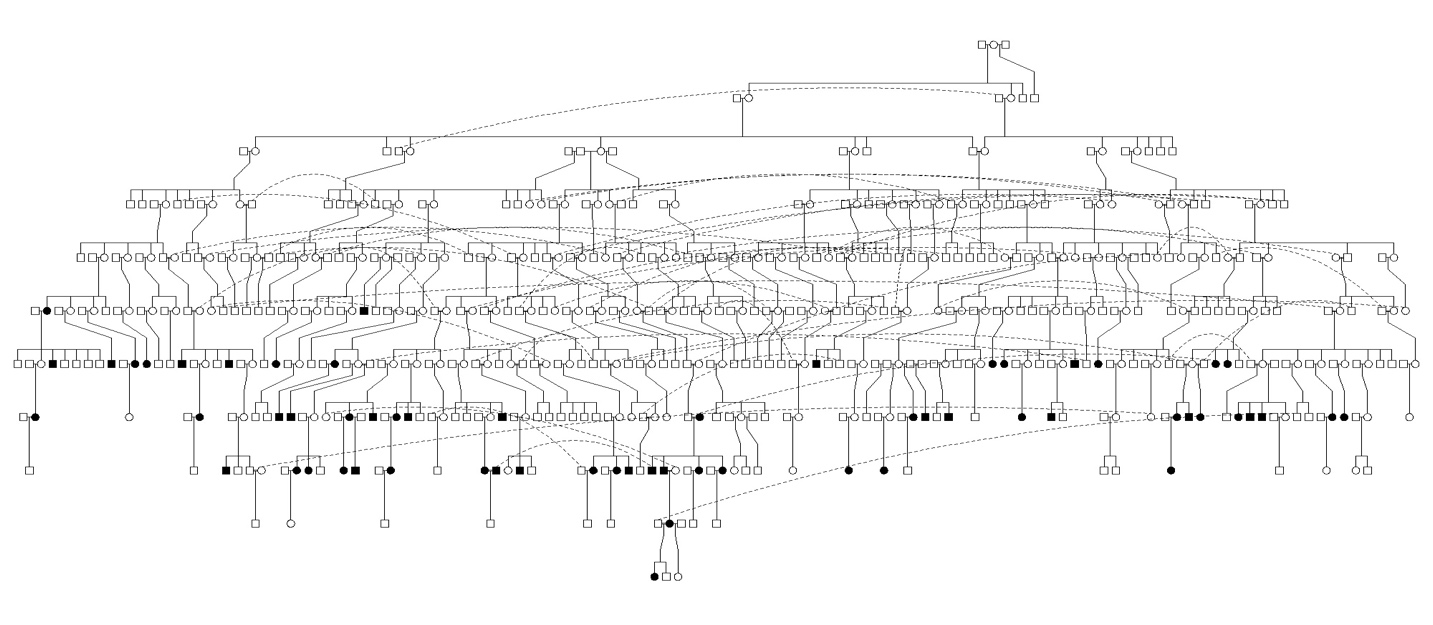

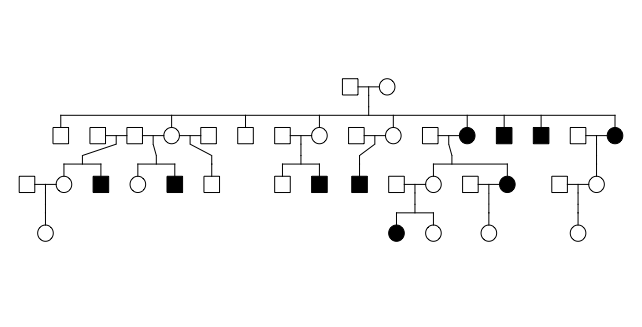

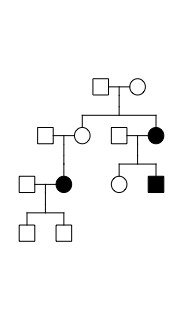

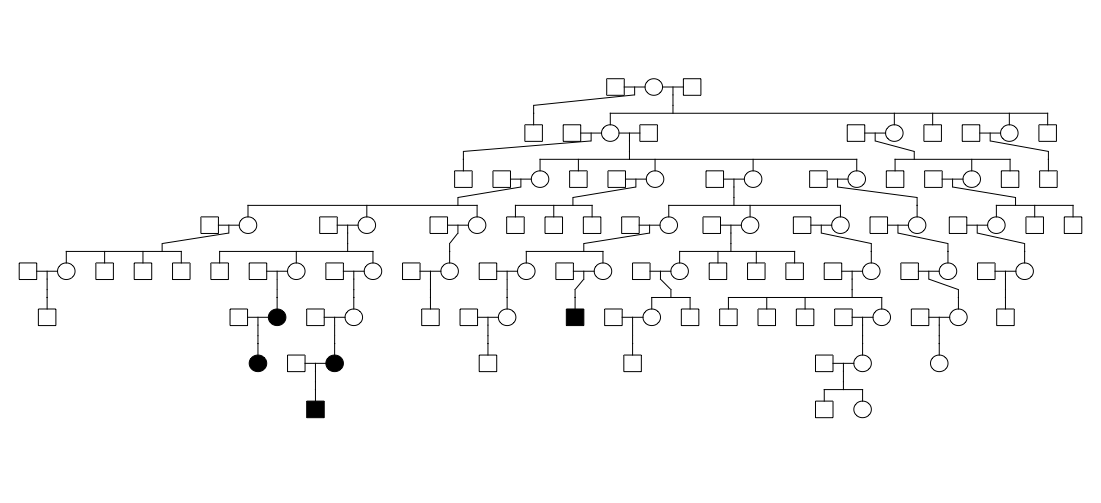

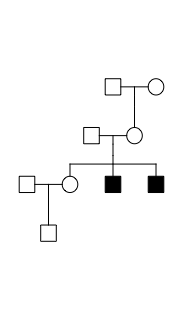

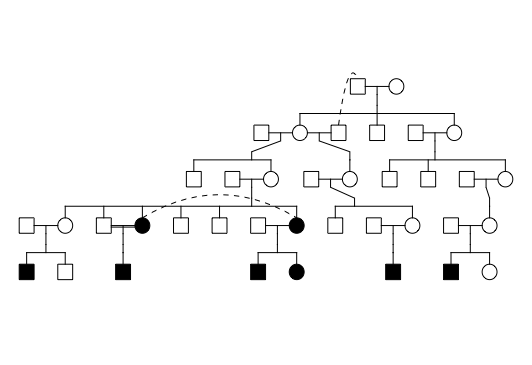

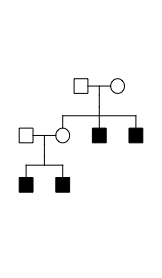

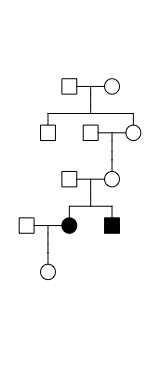

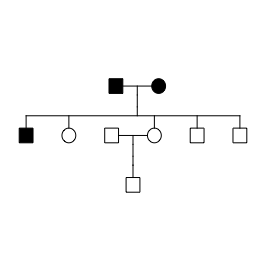

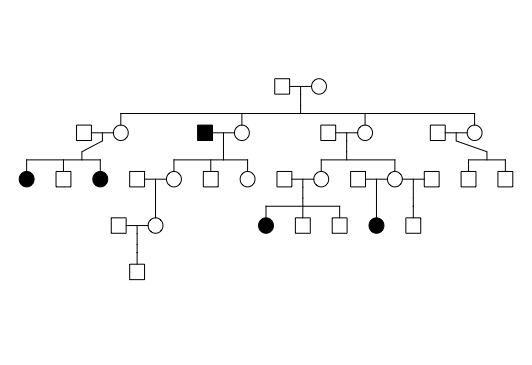

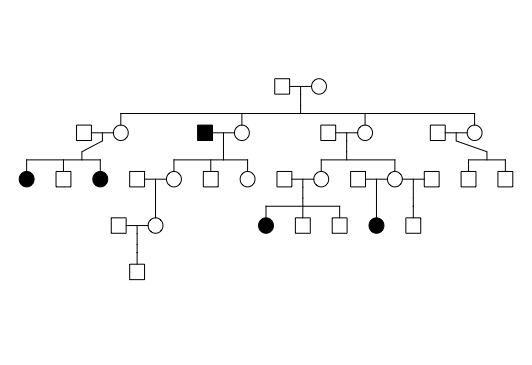

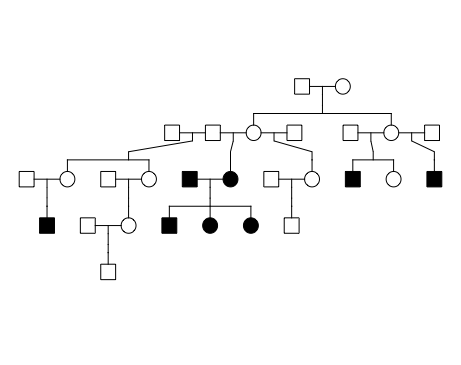

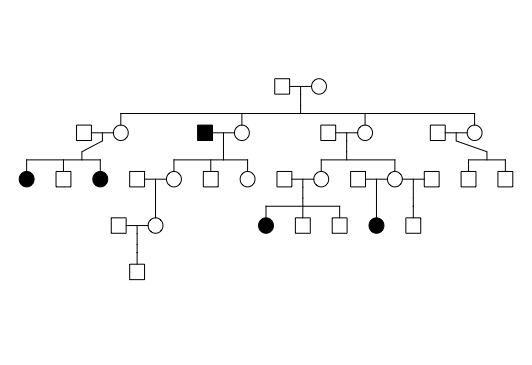

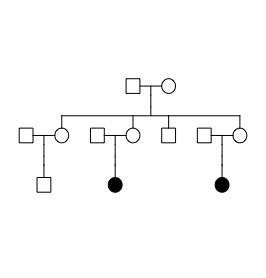

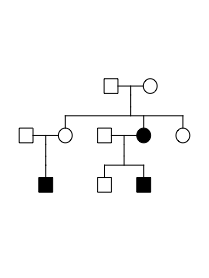

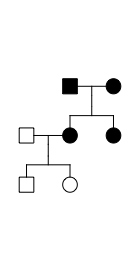

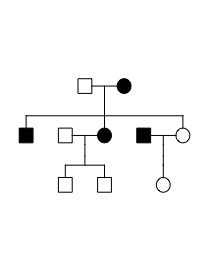

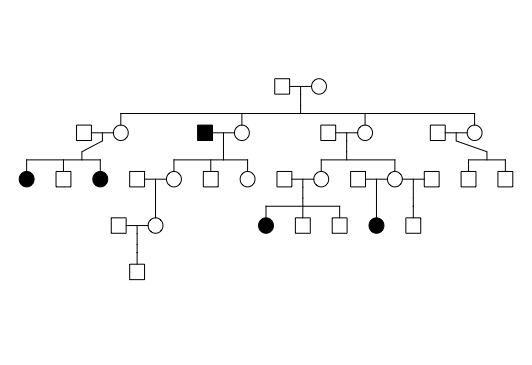

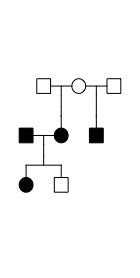

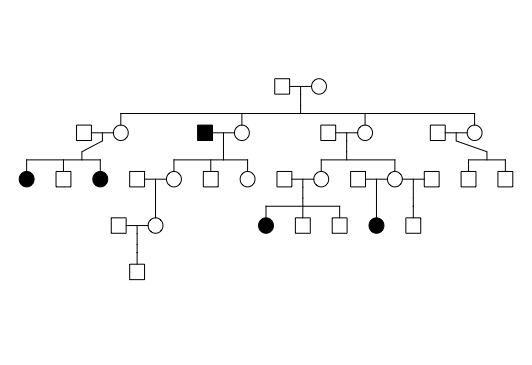

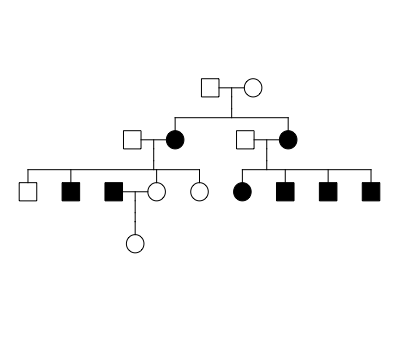

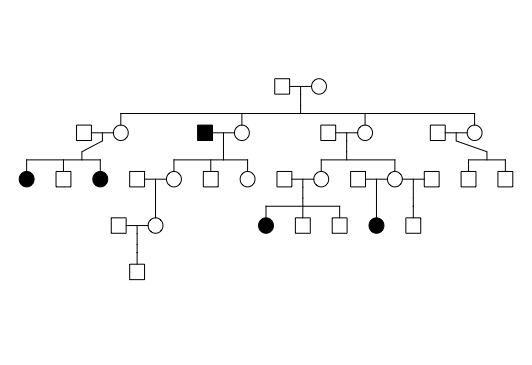

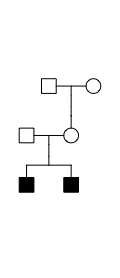

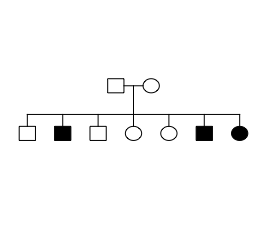

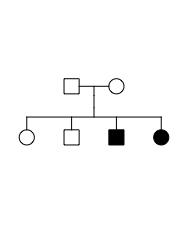

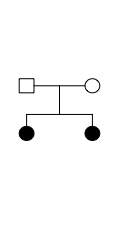

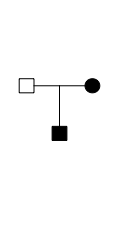

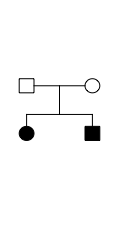

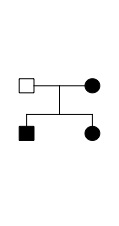

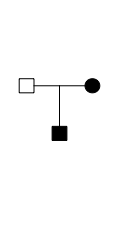

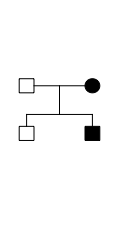

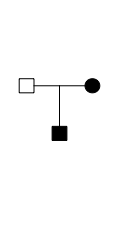

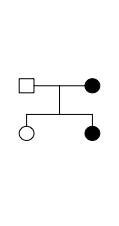

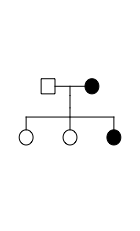

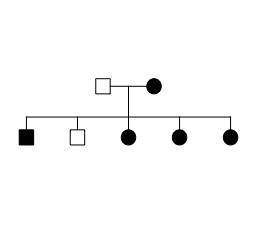

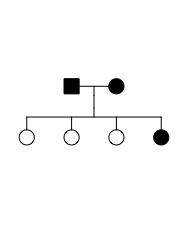

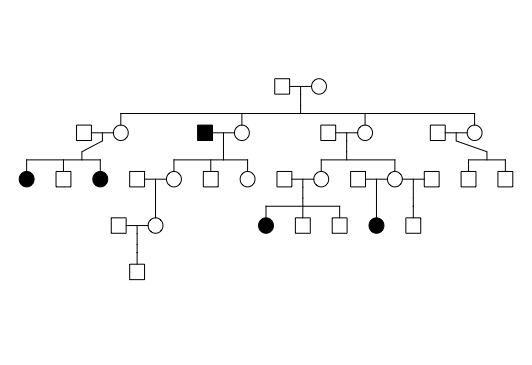

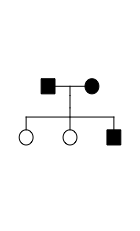

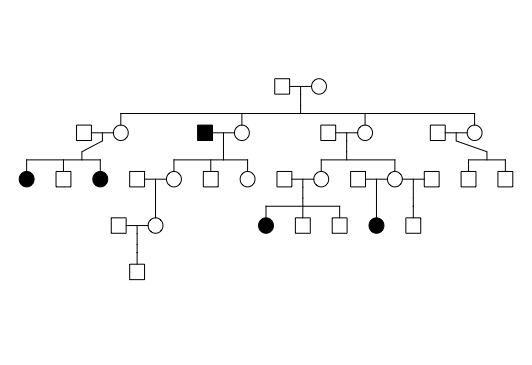

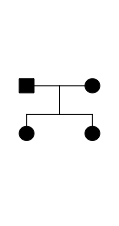

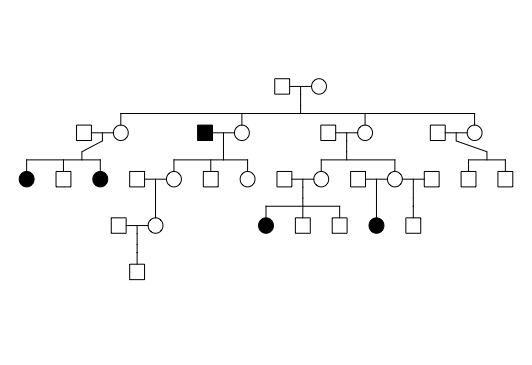

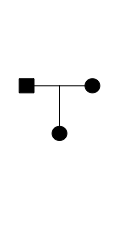

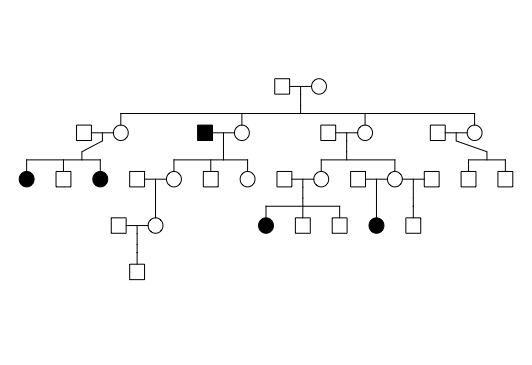

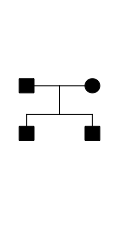

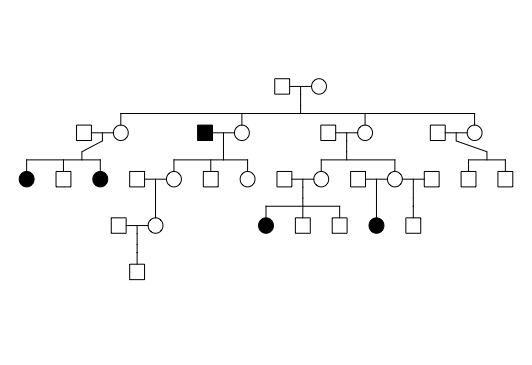

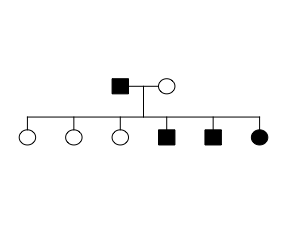

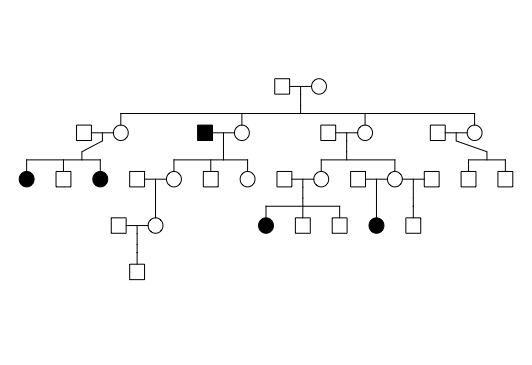

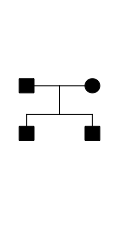

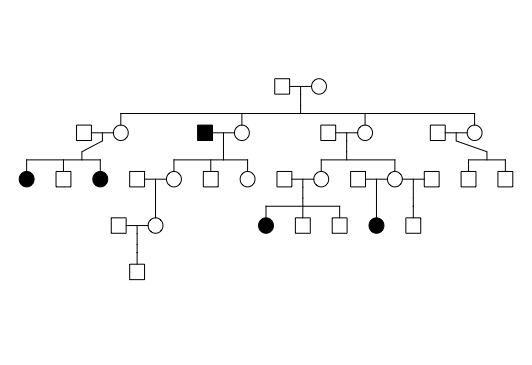

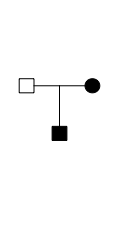

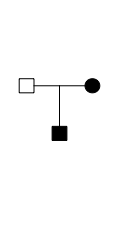

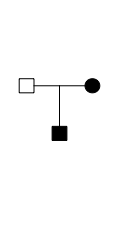

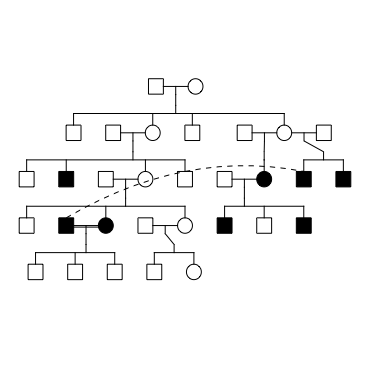

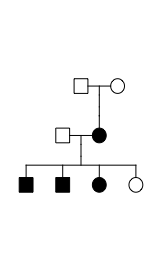

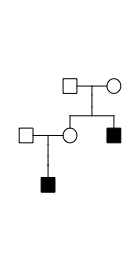

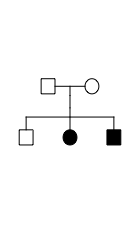


Figure adapted with permission from (25,26) a) Reconstruction of the NI pedigree based on available genealogical and genetic information (n = 1388). The pedigree spans 11 generations. b) Forty-five maternal pedigrees were included in this study. Pedigrees are derived from the NI Core Pedigree shown in (a). Individuals whose mtDNA was sequenced are shown as blackened circles (females) or squares (males)
