# Supplementary material for: Evaluating the suitability of current mitochondrial DNA interpretation guidelines for multigenerational whole mitochondrial genome comparisons
Source: J Forensic Sci. 2022 Jul 19;67(5):1766–75. doi: 10.1111/1556-4029.15097 (PMC9543078; doi:10.1111/1556-4029.15097)
Supplement: Supplementary file 2 — Figure S2 [file JFO-67-1766-s003.docx]

| a) | 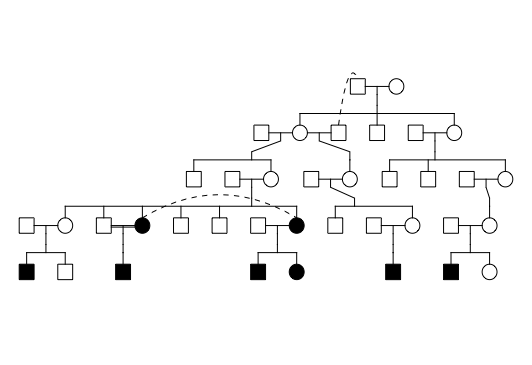 | | |
| --- | --- | --- | --- |
| b) | 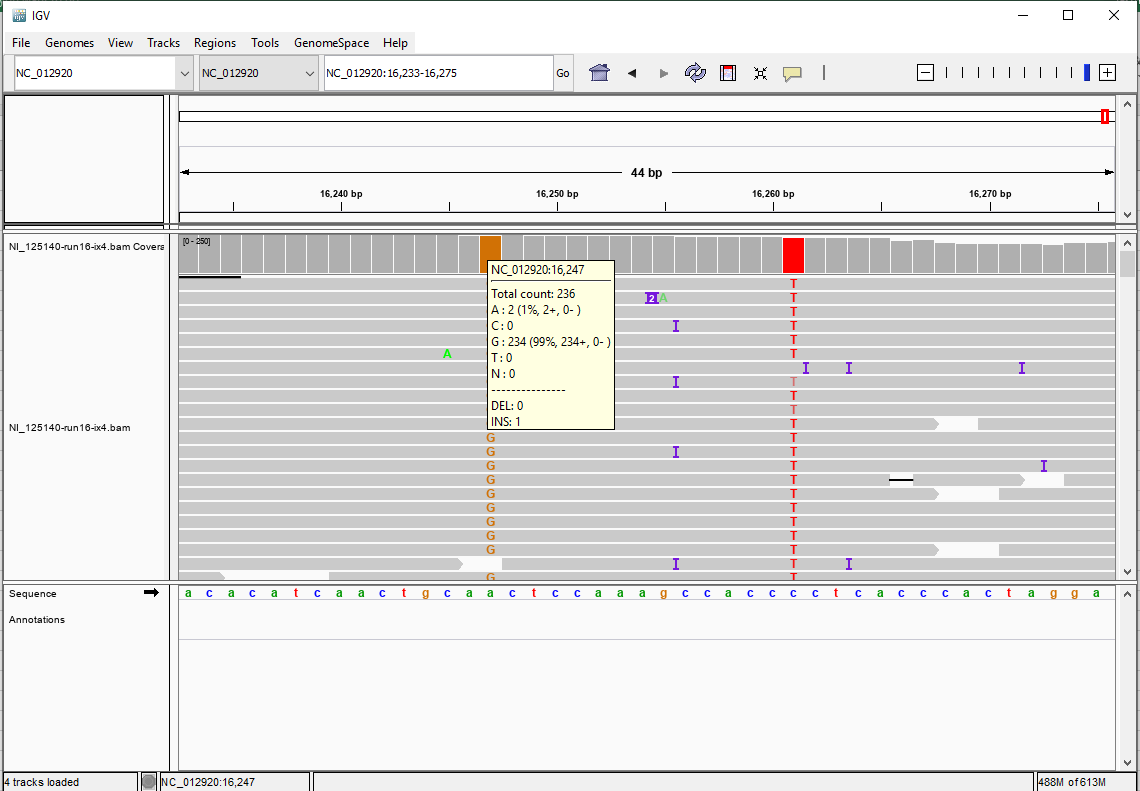 | c) | 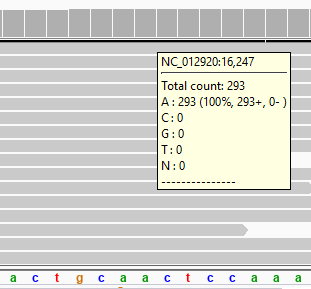 |
| d) | 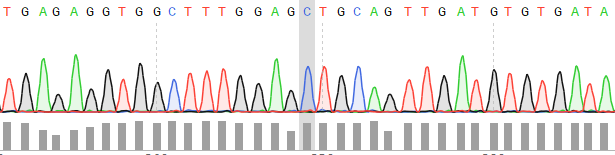 | e) | 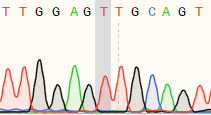 |

FIGURE S2 Identification of the A16247G variant in Family 8.

a) Pedigree for Family 8. Individuals with whole mtGenome sequences are shown as blackened circles (females) or squares (males). The red arrow indicates the individual with an A16247G variant. b and c) The IGV images of m.16247 are shown following whole mtDNA sequencing. The A16247G variant was observed in one individual from Family 8 (Figure S2b). Figure S2c shows a representative family reference sample with no A16247G variant. d and e) SS (reverse direction) of the individuals shown in Figure S2b and Figure S2c for m.16247. The m.16247 position is contoured in grey
