# Supplementary material for: Evaluating the suitability of current mitochondrial DNA interpretation guidelines for multigenerational whole mitochondrial genome comparisons
Source: J Forensic Sci. 2022 Jul 19;67(5):1766–75. doi: 10.1111/1556-4029.15097 (PMC9543078; doi:10.1111/1556-4029.15097)
Supplement: Supplementary file 3 — Figure S3 [file JFO-67-1766-s004.docx]

| a) | 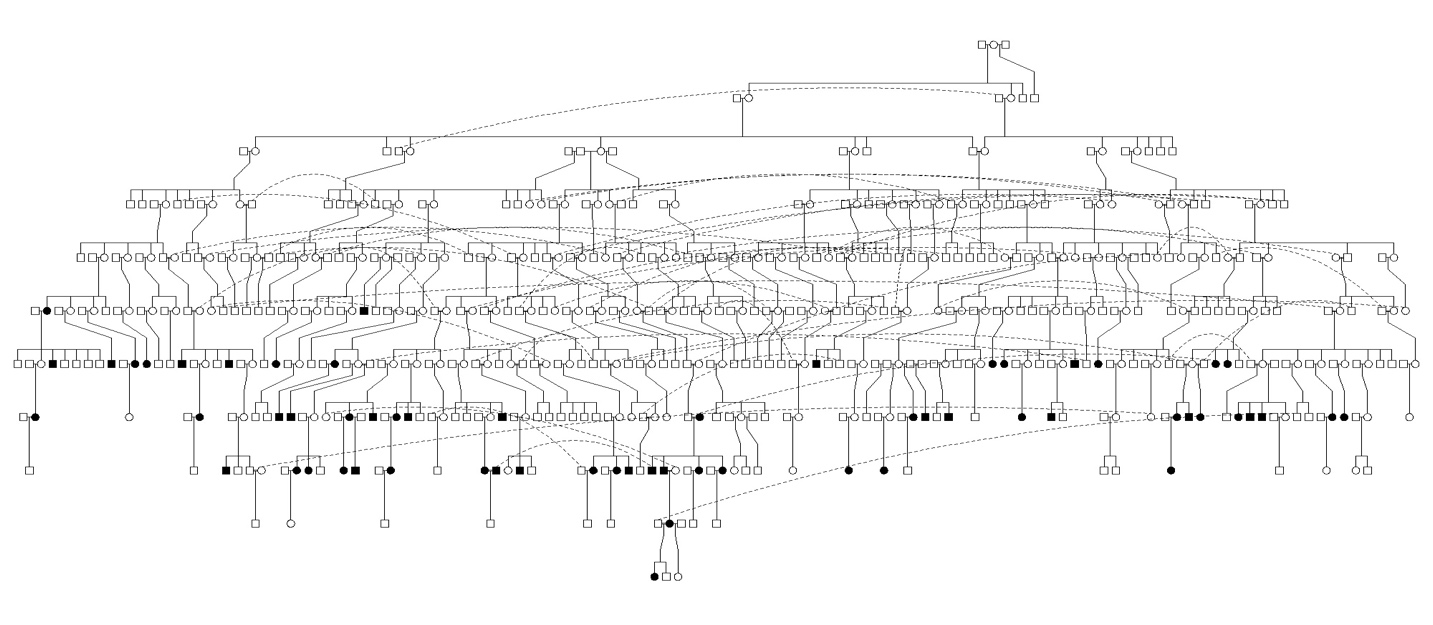 |
| --- | --- |

| b) | 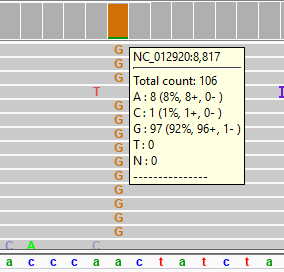 | c) | 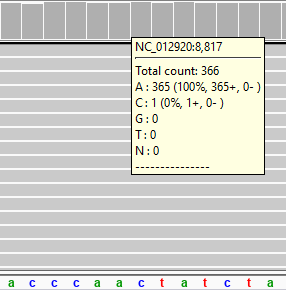 |
| --- | --- | --- | --- |
| d) | 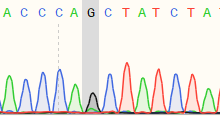 | e) | 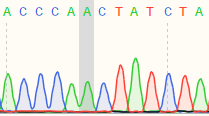 |

FIGURE S3 Identification of the A8817G variant in Family 12.

a) Pedigree for Family 12. Individuals with whole mtGenome sequences are shown as blackened circles (females) or squares (males). The red arrow indicates the individual with an A8817G variant. b and c) The IGV images of m.8817 are shown following whole mtDNA sequencing. Figure S3b shows the A8817G variant, while Figure S3c shows a representative family reference sample with no A8817G variant. d and e) SS of the individuals shown in Figure S3b and Figure S3c for m.8817. The m.8817 position is contoured in grey
