# Supplementary material for: Evaluating the suitability of current mitochondrial DNA interpretation guidelines for multigenerational whole mitochondrial genome comparisons
Source: J Forensic Sci. 2022 Jul 19;67(5):1766–75. doi: 10.1111/1556-4029.15097 (PMC9543078; doi:10.1111/1556-4029.15097)
Supplement: Supplementary file 4 — Table S1 [file JFO-67-1766-s005.docx]

# SUPPLEMENTARY TABLE S1 Amplification and sequencing primers

| Amplicon number | Amplicon size | Primer name | Primer sequence |
| --- | --- | --- | --- |
| 1 | 350 | F3 | TCACAGGTCTATCACCCTATTAACC |
|  |  | R353 | GTTTGGCAGAGATGTGTTTAAGTG |
| 2 | 535 | F362 | CAAAGAACCCTAACACCAGCCTAAC |
|  |  | R897 | GTGGCTGGCACGAAATTGACC |
| 3 | 546 | F1531 | CCCCTACGCATTTATATAGAGGAGAC |
|  |  | R2077 | GGATTTAGAGGGTTCTGTGGGC |
| 4 | 494 | F2419 | CTGTCAACCCAACACAGGCA |
|  |  | R2913 | TGGTCAAGTTATTGGATCAATTGAGT |
| 5 | 556 | F4934 | CACTCTCTCAATCTTATCCATCATAGC |
|  |  | R5490 | GGGAGATAGGTAGGAGTAGCG |
| 6 | 462 | F5828 | GTAAAAAGAGGCCTAACCCCTGTCT |
|  |  | R6290 | GTAGACTGTTCAACCTGTTCCTGCT |
| 7 | 553 | F6157 | TAATAATCGGTGCCCCCGATA |
|  |  | R6710 | TCCAAATGGTTCTTTTTTTCCGGAGTA |
| 8 | 500 | F6682 | ACTACTCCGGAAAAAAAGAACCATTT |
|  |  | R7182 | GGAAGAAAGTTAGATTTACGCCGAT |
| 9 | 495 | F7234 | CCGATGCATACACCACATGAAAC |
|  |  | R7729 | TGTTGTGAGTGTTAGGAAAAGGGCA |
| 10 | 512 | F8174 | TCTGAAATCTGTGGAGCAAACCAC |
|  |  | R8686 | AGGTTAGTTTGATTAGTCATTGTTGGG |
| 11 | 539 | F8547 | TTCATTCATTGCCCCCACAATC |
|  |  | R9086 | GGAAGGTTAATGGTTGATATTGCTAGG |
| 12 | 553 | F9228 | TATCATATAGTAAAACCCAGCCCATGA |
|  |  | R9781 | ATGCCGTCGGAAATGGTGAAG |
| 13 | 535 | F12281 | CAGCTATCCATTGGTCTTAGGC |
|  |  | R12816 | GGCGTATCATCAACTGATGAGCAAG |
| 14 | 509 | F14436 | CAGGATACTCCTCAATAGCCATCGCTG |
|  |  | R14945 | CGATTGATGAAAAGGCGGTTGAG |
| 15 | 470 | F16078 | ACAACCGCTATGTATTTCGTACATT |
|  |  | R16548 | GGGAACGTGTGGGCTATTTAGG |
