# Supplementary material for: Evaluating the suitability of current mitochondrial DNA interpretation guidelines for multigenerational whole mitochondrial genome comparisons
Source: J Forensic Sci. 2022 Jul 19;67(5):1766–75. doi: 10.1111/1556-4029.15097 (PMC9543078; doi:10.1111/1556-4029.15097)
Supplement: Supplementary file 5 — Table S2 [file JFO-67-1766-s002.docx]

# SUPPLEMENTARY TABLE S2 Haplogroup and heteroplasmy information

| Sample ID | Family ID | Haplogroup | Heteroplasmy Count  (MAF >20%) |
| --- | --- | --- | --- |
| 1 | 1 | T2c(T2c1d+152) | 1 |
| 2 | 1 | T2c(T2c1d+152) | 2 |
| 3 | 1 | T2c(T2c1d+152) | 0 |
| 4 | 1 | T2c(T2c1d+152) | 0* MAF 15%, seen in Sanger |
| 5 | 1 | T2c(T2c1d+152) | 0 |
| 6 | 1 | T2c(T2c1d+152) | 0 |
| 7 | 1 | T2c(T2c1d+152) | 0 |
| 8 | 1 | T2c(T2c1d+152) | 1 |
| 9 | 1 | T2c(T2c1d+152) | 0 |
| 10 | 1 | T2c(T2c1d+152) | 0 |
| 1 | 2 | U4a(U4a1d) | 0 |
| 2 | 2 | U4a(U4a1d) | 0 |
| 3 | 2 | U4a(U4a1d) | 0 |
| 4 | 2 | U4a(U4a1d) | 0 |
| 5 | 2 | U4a(U4a1d) | 0 |
| 6 | 2 | U4a(U4a1d) | 0 |
| 7 | 2 | U4a(U4a1d) | 0 |
| 1 | 3 | U5a(U5a1i1) | 0 |
| 2 | 3 | U5a(U5a1i1) | 0 |
| 3 | 3 | U5a(U5a1i1) | 0 |
| 1 | 4 | I2d(I2d) | 0 |
| 2 | 4 | I2d(I2d) | 1 |
| 3 | 4 | I2d(I2d) | 0 |
| 4 | 4 | I2d(I2d) | 0 |
| 5 | 4 | I2d(I2d) | 1 |
| 6 | 4 | I2d(I2d) | 1 |
| 7 | 4 | I2d(I2d) | 0* MAF 15%, seen in Sanger |
| 1 | 5 | B4a(B4a1a1m1) | 0 |
| 2 | 5 | B4a(B4a1a1m1) | 0 |
| 3 | 5 | B4a(B4a1a1m1) | 0 |
| 1 | 6 | B4a(B4a1a1a18) | 0 |
| 2 | 6 | B4a(B4a1a1a18) | 0 |
| 3 | 6 | B4a(B4a1a1a18) | 0 |
| 4 | 6 | B4a(B4a1a1a18) | 0 |
| 5 | 6 | B4a(B4a1a1a18) | 0 |
| 1 | 7 | B4a(B4a1a1m1) | 1 |
| 2 | 7 | B4a(B4a1a1m1) | 0 |
| 3 | 7 | B4a(B4a1a1m1) | 0 |
| 4 | 7 | B4a(B4a1a1m1) | 0 |
| 1 | 12 | B4a(B4a1a1m1) | 0 |
| 2 | 12 | B4a(B4a1a1m1) | 0 |
| 3 | 12 | B4a(B4a1a1m1) | 0 |
| 4 | 12 | B4a(B4a1a1m1) | 0 |
| 5 | 12 | B4a(B4a1a1m1) | 0 |
| 6 | 12 | B4a(B4a1a1m1) | 0 |
| 7 | 12 | B4a(B4a1a1m1) | 1 |
| 8 | 12 | B4a(B4a1a1m1) | 1 |
| 9 | 12 | B4a(B4a1a1m1) | 0 |
| 10 | 12 | B4a(B4a1a1m1) | 0 |
| 11 | 12 | B4a(B4a1a1m1) | 0 |
| 12 | 12 | B4a(B4a1a1m1) | 0 |
| 13 | 12 | B4a(B4a1a1m1) | 0 |
| 14 | 12 | B4a(B4a1a1m1) | 0 |
| 15 | 12 | B4a(B4a1a1m1) | 0 |
| 16 | 12 | B4a(B4a1a1m1) | 0 |
| 17 | 12 | B4a(B4a1a1m1) | 0 |
| 18 | 12 | B4a(B4a1a1m1) | 0 |
| 19 | 12 | B4a(B4a1a1m1) | 0 |
| 20 | 12 | B4a(B4a1a1m1) | 0 |
| 21 | 12 | B4a(B4a1a1m1) | 0 |
| 22 | 12 | B4a(B4a1a1m1) | 0 |
| 23 | 12 | B4a(B4a1a1m1) | 0 |
| 24 | 12 | B4a(B4a1a1m1) | 0 |
| 25 | 12 | B4a(B4a1a1m1) | 0 |
| 26 | 12 | B4a(B4a1a1m1) | 0 |
| 27 | 12 | B4a(B4a1a1m1) | 0 |
| 28 | 12 | B4a(B4a1a1m1) | 0 |
| 29 | 12 | B4a(B4a1a1m1) | 0 |
| 30 | 12 | B4a(B4a1a1m1) | 0 |
| 31 | 12 | B4a(B4a1a1m1) | 0 |
| 32 | 12 | B4a(B4a1a1m1) | 0 |
| 33 | 12 | B4a(B4a1a1m1) | 0 |
| 34 | 12 | B4a(B4a1a1m1) | 0 |
| 35 | 12 | B4a(B4a1a1m1) | 0 |
| 36 | 12 | B4a(B4a1a1m1) | 0 |
| 37 | 12 | B4a(B4a1a1m1) | 0 |
| 38 | 12 | B4a(B4a1a1m1) | 0 |
| 39 | 12 | B4a(B4a1a1m1) | 0 |
| 40 | 12 | B4a(B4a1a1m1) | 0 |
| 41 | 12 | B4a(B4a1a1m1) | 0 |
| 42 | 12 | B4a(B4a1a1m1) | 0 |
| 43 | 12 | B4a(B4a1a1m1) | 0 |
| 44 | 12 | B4a(B4a1a1m1) | 0 |
| 45 | 12 | B4a(B4a1a1m1) | 0 |
| 46 | 12 | B4a(B4a1a1m1) | 0 |
| 47 | 12 | B4a(B4a1a1m1) | 0 |
| 48 | 12 | B4a(B4a1a1m1) | 0 |
| 49 | 12 | B4a(B4a1a1m1) | 0 |
| 50 | 12 | B4a(B4a1a1m1) | 0 |
| 51 | 12 | B4a(B4a1a1m1) | 0 |
| 52 | 12 | B4a(B4a1a1m1) | 0 |
| 53 | 12 | B4a(B4a1a1m1) | 0 |
| 54 | 12 | B4a(B4a1a1m1) | 0 |
| 55 | 12 | B4a(B4a1a1m1) | 0 |
| 56 | 12 | B4a(B4a1a1m1) | 0 |
| 57 | 12 | B4a(B4a1a1m1) | 0 |
| 58 | 12 | B4a(B4a1a1m1) | 0 |
| 59 | 12 | B4a(B4a1a1m1) | 0 |
| 1 | 9 | B4a(B4a1a1m1) | 0 |
| 2 | 9 | B4a(B4a1a1m1) | 0 |
| 3 | 9 | B4a(B4a1a1m1) | 0 |
| 4 | 9 | B4a(B4a1a1m1) | 0 |
| 5 | 9 | B4a(B4a1a1m1) | 0 |
| 6 | 9 | B4a(B4a1a1m1) | 0 |
| 7 | 9 | B4a(B4a1a1m1) | 0 |
| 8 | 9 | B4a(B4a1a1m1) | 0 |
| 9 | 9 | B4a(B4a1a1m1) | 0 |
| 10 | 9 | B4a(B4a1a1m1) | 0 |
| 11 | 9 | B4a(B4a1a1m1) | 0 |
| 12 | 9 | B4a(B4a1a1m1) | 0 |
| 13 | 9 | B4a(B4a1a1m1) | 0 |
| 14 | 9 | B4a(B4a1a1m1) | 0 |
| 15 | 9 | B4a(B4a1a1m1) | 0 |
| 16 | 9 | B4a(B4a1a1m1) | 0 |
| 17 | 9 | B4a(B4a1a1m1) | 0 |
| 18 | 9 | B4a(B4a1a1m1) | 0 |
| 19 | 9 | B4a(B4a1a1m1) | 0 |
| 20 | 9 | B4a(B4a1a1m1) | 0 |
| 21 | 9 | B4a(B4a1a1m1) | 0 |
| 22 | 9 | B4a(B4a1a1m1) | 0 |
| 23 | 9 | B4a(B4a1a1m1) | 0 |
| 24 | 9 | B4a(B4a1a1m1) | 0 |
| 25 | 9 | B4a(B4a1a1m1) | 0 |
| 26 | 9 | B4a(B4a1a1m1) | 0 |
| 27 | 9 | B4a(B4a1a1m1) | 0 |
| 28 | 9 | B4a(B4a1a1m1) | 0 |
| 29 | 9 | B4a(B4a1a1m1) | 0 |
| 1 | 10 | B4a(B4a1a1m1) | 0 |
| 2 | 10 | B4a(B4a1a1m1) | 0 |
| 1 | 11 | U5a(U5a1i1) | 0 |
| 2 | 11 | U5a(U5a1i1) | 0 |
| 3 | 11 | U5a(U5a1i1) | 0 |
| 4 | 11 | U5a(U5a1i1) | 0 |
| 1 | 8 | B4a(B4a1a1m1) | 0 |
| 2 | 8 | B4a(B4a1a1m1) | 0 |
| 3 | 8 | B4a(B4a1a1m1) | 0 |
| 4 | 8 | B4a(B4a1a1m1) | 0 |
| 5 | 8 | B4a(B4a1a1m1) | 0 |
| 6 | 8 | B4a(B4a1a1m1) | 0 |
| 7 | 8 | B4a(B4a1a1m1) | 0 |
| 1 | 13 | B4a(B4a1a1h) | 0 |
| 2 | 13 | B4a(B4a1a1h) | 0 |
| 3 | 13 | B4a(B4a1a1h) | 0 |
| 4 | 13 | B4a(B4a1a1h) | 0 |
| 5 | 13 | B4a(B4a1a1h) | 0 |
| 6 | 13 | B4a(B4a1a1h) | 0 |
| 7 | 13 | B4a(B4a1a1h) | 0 |
| 1 | 14 | J1c(J1c10) | 1 |
| 2 | 14 | J1c(J1c10) | 1 |
| 3 | 14 | J1c(J1c10) | 1 |
| 4 | 14 | J1c(J1c10) | 1 |
| 1 | 15 | M42a(M42a) | 0 |
| 2 | 15 | M42a(M42a) | 0 |
| 1 | 16 | B4a(B4a1a1m1) | 0 |
| 2 | 16 | B4a(B4a1a1m1) | 0 |
| 1 | 17 | B4a(B4a1a1m1) | 0 |
| 2 | 17 | B4a(B4a1a1m1) | 0 |
| 1 | 18 | V(V) | 0 |
| 2 | 18 | V(V) | 0 |
| 3 | 18 | V(V) | 0 |
| 1 | 19 | H1b(H1bz) | 0 |
| 2 | 19 | H1b(H1bz) | 0 |
| 1 | 20 | V3c(V3c) | 0 |
| 2 | 20 | V3c(V3c) | 0 |
| 3 | 20 | V3c(V3c) | 0 |
| 1 | 21 | H1c(H1c1) | 0 |
| 2 | 21 | H1c(H1c1) | 0 |
| 1 | 22 | H17(H17) | 0 |
| 2 | 22 | H17(H17) | 0 |
| 1 | 23 | J1c(J1c2m1) | 0 |
| 2 | 23 | J1c(J1c2m1) | 0 |
| 1 | 24 | X2b(X2b11) | 0 |
| 2 | 24 | X2b(X2b11) | 1 |
| 1 | 25 | W1(W1) | 0 |
| 2 | 25 | W1(W1) | 0 |
| 3 | 25 | W1(W1) | 0 |
| 1 | 26 | H3i(H3i) | 0 |
| 2 | 26 | H3i(H3i) | 0 |
| 1 | 27 | U5b(U5b2a2a) | 0 |
| 2 | 27 | U5b(U5b2a2a) | 0 |
| 1 | 28 | K1a(K1a3a) | 0 |
| 2 | 28 | K1a(K1a3a) | 0 |
| 1 | 29 | U5a(U5a1a1+152) | 0 |
| 2 | 29 | U5a(U5a1a1+152) | 0 |
| 1 | 30 | H47a(H47a) | 0 |
| 2 | 30 | H47a(H47a) | 0 |
| 1 | 31 | H13a(H13a1a) | 0 |
| 2 | 31 | H13a(H13a1a) | 0 |
| 3 | 31 | H13a(H13a1a) | 0 |
| 1 | 32 | P1(P1) | 0 |
| 2 | 32 | P1(P1) | 0 |
| 1 | 33 | J1c(J1c3) | 0 |
| 2 | 33 | J1c(J1c3) | 0 |
| 1 | 34 | J1c(J1c5) | 0 |
| 2 | 34 | J1c(J1c5) | 0 |
| 1 | 35 | B4a(B4a1a1) | 0 |
| 2 | 35 | B4a(B4a1a1) | 0 |
| 1 | 36 | X2b(X2b11) | 0 |
| 2 | 36 | X2b(X2b11) | 0 |
| 1 | 37 | T1a(T1a1g) | 0 |
| 2 | 37 | T1a(T1a1g) | 0 |
| 3 | 37 | T1a(T1a1g) | 0 |
| 1 | 38 | H56b(H56b) | 0 |
| 2 | 38 | H56b(H56b) | 0 |
| 1 | 39 | B4a(B4a1a1) | 0 |
| 2 | 39 | B4a(B4a1a1) | 0 |
| 1 | 40 | U5a(U5a1h) | 0 |
| 2 | 40 | U5a(U5a1h) | 0 |
| 3 | 40 | U5a(U5a1h) | 0 |
| 1 | 41 | H55(H55) | 0 |
| 2 | 41 | H55(H55) | 0 |
| 3 | 41 | H55(H55) | 0 |
| 1 | 42 | W1(W1) | 0 |
| 2 | 42 | W1(W1) | 0 |
| 3 | 42 | W1(W1) | 0 |
| 1 | 43 | K2b(K2b1a3) | 0 |
| 2 | 43 | K2b(K2b1a3) | 0 |
| 3 | 43 | K2b(K2b1a3) | 1 |
| 4 | 43 | K2b(K2b1a3) | 1 |
| 5 | 43 | K2b(K2b1a3) | 0* MAF 12%, seen in Sanger |
| 1 | 44 | H6a(H6a1a) | 0 |
| 2 | 44 | H6a(H6a1a) | 0 |
| 3 | 45 | X2b(X2b+226) | 0 |
| 4 | 45 | X2b(X2b+226) | 0 |
| 5 | 45 | X2b(X2b+226) | 0 |
